# Supplementary material for: Isolation of Methane Enriched Bacterial Communities and Application as Wheat Biofertilizer under Drought Conditions: An Environmental Contribution
Source: Plants (Basel). 2023 Jun 29;12(13):2487. doi: 10.3390/plants12132487 (PMC10347144; doi:10.3390/plants12132487)
Supplement: Supplementary file 1 [file plants-12-02487-s001.zip › plants-2416317-SI.pdf]

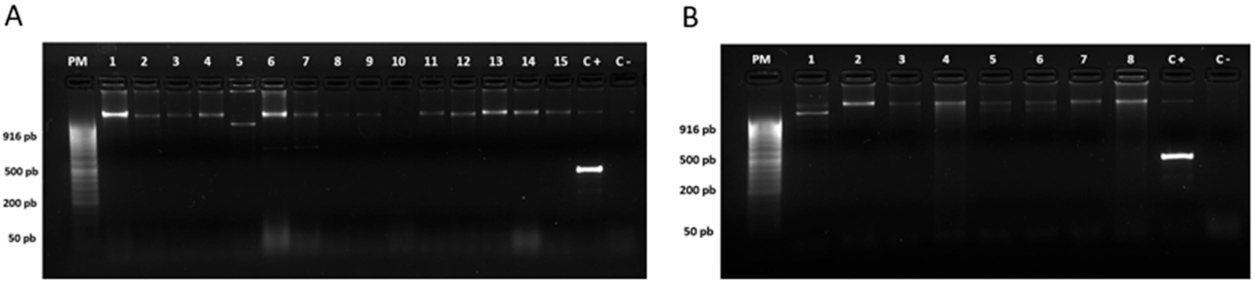


**Supplementary Figure S1:** Amplification of *pmo*A gene from non-methanotrophs isolates DNA by PCR.

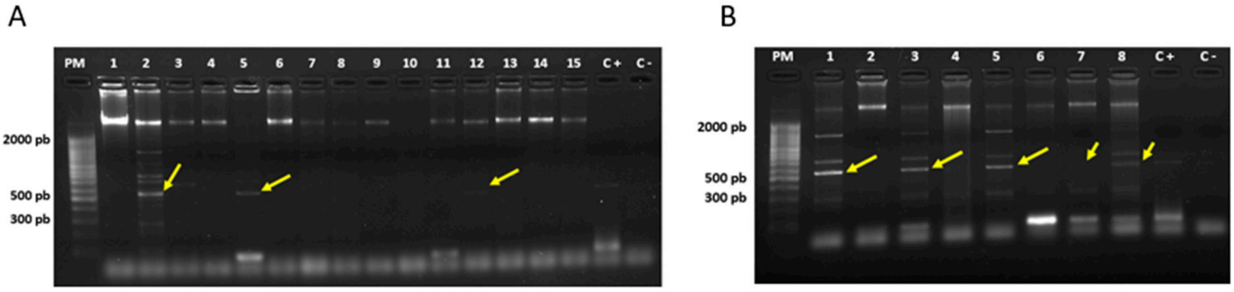


**Supplementary Figure S2:** Amplification of *mxa*F gene from non-methanotrophs isolates DNA by PCR.

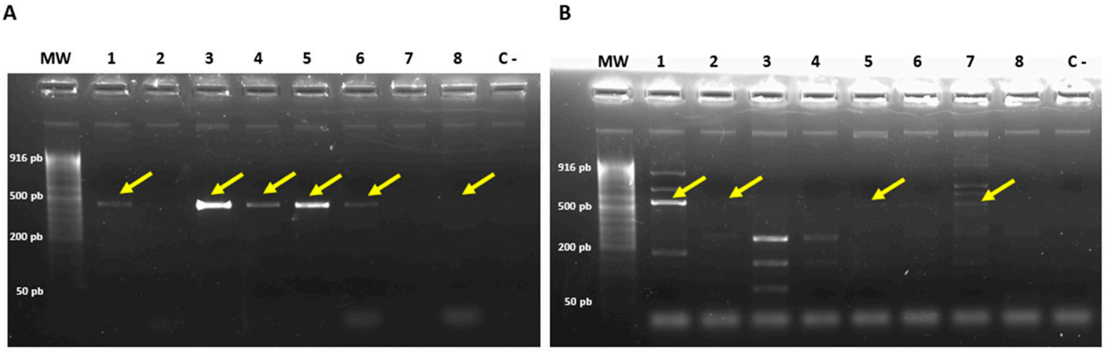


**Supplementary Figure S3:** Amplification of communities of methanotrophs and associated microorganisms DNA by PCR using specific oligonucleotides for *pmo*A (A) and *mxa*F (B) genes.
